# Consumers’ Attitude to Consumption of Rabbit Meat in Eight Countries Depending on the Production Method and Its Purchase Form

**DOI:** 10.3390/foods9050654

**Published:** 2020-05-19

**Authors:** Katalin Szendrő, Eszter Szabó-Szentgróti, Orsolya Szigeti

**Affiliations:** Institute of Marketing and Management, Kaposvár University, H-7400 Kaposvár, Hungary; szentgroti.eszter@ke.hu (E.S.-S.); szigeti.orsolya@ke.hu (O.S.)

**Keywords:** rabbit, meat, consumption, attitude, production method, purchase

## Abstract

**Simple Summary:**

The aim of the study was to investigate the consumers’ attitude to their preference of rabbit meat in eight countries depending on the production method and its purchase form. Consumers’ attitude was evaluated in Spain, Italy, France, Poland, Hungary, China, Brazil and Mexico based on the respondents’ opinion regarding origin, production conditions, slaughtering methods and breed. Results revealed that breed was an inessential factor. The origin of rabbits was the most important in Italy, France, Hungary and Brazil (in the latter country the slaughtering method and the level of processing were also highly valued), feeding of animals received the highest scores in Spain, Poland and Mexico (in the latter also the slaughtering method), while the level of processing was the foremost factor in China. The preference of fresh meat was the highest in Spain, France and Mexico, and that of frozen in Brazil and Mexico. The highest preference of a whole carcass was found in France and Mexico. The thigh was mostly favored in France and that of the loin in Mexico. The prepared meat products (roasted, smoked and semi-finished) were the most popular in Mexico. It can be concluded the preference of respondents depended on the country.

**Abstract:**

The study’s aim was to investigate the consumers’ attitude to their preference of rabbit meat in eight countries depending on the production method and its purchase form. In Spain and China almost all factors got low scores. High scores (above 4 out of 5) for origin were found in Italy, France, Poland, Hungary and Brazil. The importance of feeding was highlighted in Italy, Poland, Hungary and Mexico. High values were received for housing conditions in Italy, Poland, Hungary and Mexico. The level of processing was the most important in China and Brazil, while the slaughtering method was considered the most important in Brazil and Mexico. Breed received the lowest score in almost all countries. The preference of fresh meat was the highest (above 50%) in Spain, France and Mexico, and that of frozen in Brazil and Mexico (about 20%). The highest preference for a whole carcass (above 50%) was given by the respondents in France and Mexico. Thigh was mostly preferred in France whereas consumers preferred loin in Mexico. Roasted, smoked and semi-finished forms were mostly favored in Mexico. It can be concluded that the preference of respondents depended on the country. Preferences were different among the Mediterranean countries, and also Latin American countries.

## 1. Introduction

Although rabbit meat offers excellent nutritive and dietetic properties [1], its consumption accounts for less than 3% of all meats consumed in the EU [2]. It is healthy due to its high contents of polyunsaturated fatty acids, proteins and essential amino acids, moderately high energy values, low fat and low cholesterol levels, important source of B vitamins (mainly B12 vitamin), it has low sodium content, but it is rich in phosphorus and its selenium level depends on the supplementation [3,4].

Compared to the amount of meats produced from different farm animals, rabbit meat plays a minor role. In China the production and consumption are increasing; the proportion of China to the world production is about 60% [5]. Although France, Italy and Spain have a long tradition in rabbit meat production and consumption, a decreasing trend can be detected in these Mediterranean countries. According to the database of FAOSTAT [6] the rabbit meat production increased from 370,000 to 865,477 t/yr in China (+134%), from 4160 to 4483 t/yr in Mexico (+8%), from 42,174 to 43,109 t/yr in Italy (+2%; however, according to Trocino et al. [7] it decreased to 29,000 t in 2017), and decreased from 3300 to 3000 t/yr in Poland (−17%), from 73,367 to 43,886 t/yr in France (−33%), from 2100 to 1194 t/yr in Brazil (−43%), from 103,596 to 55,824 t/yr in Spain (−46%) and from 14,000 to 5641 t/yr in Hungary (−60%) between 2000 and 2018.

The change in production shows the tendency of consumption. However, when the factors influencing rabbit meat consumption are examined, the yearly rabbit meat consumption per capita is more informative than the countries’ production, export and import. The global yearly rabbit meat consumption per capita represents only a small part (0.19 kg) [8] of total meat consumption. It is 0.51 kg in the EU [8], 1.09 kg in Spain [8], 0.91 kg in Italy [8], 0.75 kg in France [8], 0.1 kg in Hungary [9], 0.3 kg in China [10], 0.2 kg in Mexico [11] and 0.08 kg in Brazil [11].

There has been an increasing interest in animal housing and the associated welfare standards in several countries. Welfare is a key point in the consumer’s preferences and meat choices. This attitude is even more relevant for the rabbit as it is also a pet animal [12]. However, the direct relationship between the consumer and animal production is very weak; their knowledge about it is limited. It is a great challenge for the Mediterranean countries to change the housing systems as recommended by the animal rights activists, which is mostly accepted by consumers, e.g., pens for group rearing instead of bicellular cages for growing rabbits in Italy [7] or changing the traditional cages of rabbit does for cages with an elevated platform. It is a high expense, thus it will result in further reduction in production, as many farmers will not be able to finance it. At the same time in Poland small farms are dominant [13]. In Hungary, due to the fact that most rabbit meat is exported to Switzerland and Germany (where there is a high expectation of animal welfare), this change in the housing systems has already been done [14].

The consumer preferences for rabbit meat have been investigated by some researchers: in Spain [15,16,17,18,19,20,21,22], in France [23], in Hungary [24,25,26], in Poland [27], in Romania [28], in China [10], in Indonesia [29], in Mexico [30], in the USA [31,32,33], in South Africa [34,35], in Nigeria [36], in Kenya [37] and in Tanzania [38]. As an example, some studies are presented on what authors from different countries have examined in relation to rabbit meat consumption. When a topic was examined in the particular country that was also included in the present research, it was used in the discussion.

- Hungary [24]: connection with rabbit production, opinion on rabbit meat consumption (causes of rejection), opinion on rabbit meat, frequency of consumption, place and form of purchasing, opinion on prices, information about cooking recipes and effects that could increase consumption frequency.
- Mexico [30]: frequency of consumption, selling points for rabbit meat, causes of purchasing, opinion of the flavor of rabbit meat and causes of rejection.
- Spain [17]: consumption of rabbit meat (type), opinion on rabbit meat consumption (causes of rejection), preferred meat, ownership of rabbits as pets and hunters in the family. Kallas and Gil [16]: origin (foreign, Spain, Catalonia), format (boneless, pieced and entire) and brand (unbranded, commercial brand and quality brand). Escribá-Pérez et al. [20]: opinion about rabbit meat consumption (causes of rejection), frequency of meat consumption (beef, chicken, pork and rabbit), benefits provided by rabbit meat and types of rabbit meat presentations.
- France [23]: frequency of consumption, place of consumption, causes of not eating more rabbit, time and the most important criteria of purchasing.

In these papers only one country was examined. The advantage of the present study is that the same questions were answered by respondents from more countries, and thus the opinions of consumers were objectively comparable. However, most of the respondents had a higher level of education, so the results of the study primarily reflect their opinions.

From a product development and marketing point of view, it is important to become familiar with the expectations and preferences of consumers in general and depending on their gender, age and also their income. Important information can be obtained about the knowledge and expectations of consumers in other countries, especially if a company wants to export rabbit meat there. This is why the aim of the present study was to investigate the consumers’ attitude to the preference of rabbit meat in eight countries depending on the production method and its purchase form.

## 2. Materials and Methods 

The global consumer study was conducted in 2018. The survey consisted of 28 structured questions, asking respondents—among other questions—about their opinion on the importance of origin, production condition and slaughtering method of rabbits, as well as purchase and preparation forms of rabbit meat. Among non-probability sampling techniques, snowball sampling of data collection was used meaning that the structured survey was given to an initial group of respondents (those who used the Internet) selected randomly. Respondents were encouraged to locate other members of the target population whom they know, i.e., friends, relatives, colleagues, etc. Since in the case of China, the online questionnaire reached only a few people directly, we asked a colleague for help who was primarily able to engage university students and staff. Multiple responses were excluded since the system allowed only one response/IP address. The total number of responses was 2205 (in Hungary: 420, in Spain: 227, in China: 201, in Italy: 242, in Poland: 198, in France: 67, in Brazil: 360, in Mexico: 60 and other counties: 430. Subgroups were formed based on the background information: gender, age and income. The numbers of respondents are shown in Table 1 and in the other tables in the Results and Discussion. 

Production conditions and slaughtering methods of rabbits (origin, breed, feeding, housing, slaughtering and level of processing) and purchasing form of rabbit meat (14 groups from fresh or frozen products to different prepared forms) were analyzed. Respondents ranked the origin, breed, feeding method, housing system, slaughtering method and level of processing on a five-level scale based on their importance (1: not at all important, 5: extremely important). In the question asking about the preferred form of purchase, multiple response options were allowed, so the “*n*” could be more than the number of total responses, and the total percentage more than 100%.

In the questionnaire, we emphasized that the survey was anonymous and asked for their consent in the first point to publish the results in a scientific paper.

### Statistical Analysis 

Only faultless questionnaires were evaluated. The questionnaire was evaluated with a one-way ANOVA using SPSS 10.0 software:

Yij = μ + Vi + eij(1)

where: μ = general mean, Vi = effect of the variables (I = 1–2) and eij = random error. Frequency distributions, cross tables (for determining the relation of a variable to the background variables and to other involved variables) were used in the evaluation of the questionnaire. In addition, mean calculations and significance analysis (Chi^2^-probe) were performed. For background variables, respondents whose proportion did not reach 3% were excluded from the analyses due to the low number of items.

## 3. Results and Discussion

The purchase decision was influenced by several factors. In the present paper two main factors were investigated: production condition and slaughtering method of rabbits as well as preferred purchasing form of rabbit meat. When data were analyzed, we had to keep in mind that they represented a group of people, i.e., mainly students and highly educated persons who made up approximately 90% of all respondents.

### 3.1. Production Methods and Slaughtering

**Figure 1:**
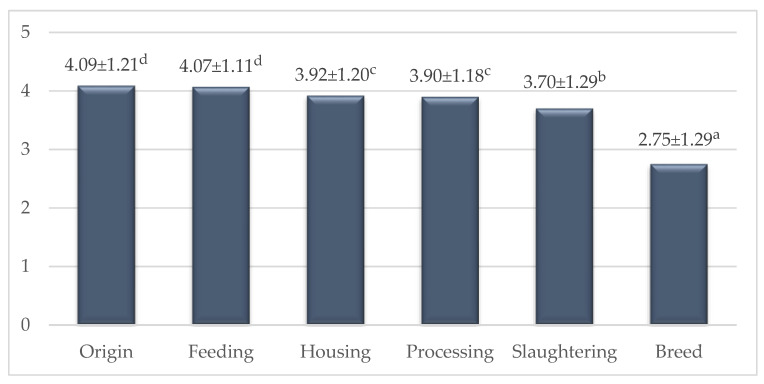
Effect of the production method and purchase form on buying decision (on a 1–5 scale). Mean ± SD; ^a–d^ means with different superscripts differ (*p* < 0.05).

The effect of origin, production system and slaughtering method on purchase decision is shown in Figure 1. Significant differences were found among different factors (*p* < 0.001). A mean score above 4 shows that the factor was very important. That is, respondents highly valued the origin of rabbit and the feeding method. Origin was not important for the quality of meat, but it indicates a commitment to local (or national) products. The Spanish surveys show this clearly [15,16]. In their studies the most important origin attribute was the Catalonian rabbit, followed by Spanish rabbits, while imported (foreign) rabbit meat was less preferred.

The importance of feeding had a similar score to that of the origin (Figure 1). Indeed, one of the most important factors in terms of meat quality could be the feeding [1,3], which can be related to health.

The housing and level of processing received lower points by 0.15–0.19 (*p* < 0.05) than those of origin and feeding (Figure 1). The demand for welfare-friendly animal products has increased in recent years mainly in EU countries [39]. Currently, housing and animal welfare are the focus of consumer interest. These can significantly influence consumers’ intention to buy and consume any animal product. As a result, this factor was rated close to 4.

The level of processing is very important for people who cook rabbit food. Some people like cooking traditional food, but there are more and more people, especially young, who spend less time in the kitchen and prefer buying semi-prepared and ready-made products [40,41]. As the value (3.90) in Figure 1 shows, this was an important aspect for the respondents. Therefore, in the second part of this paper we would deal with this topic in more detail.

The slaughtering method could be in close connection with welfare; this is why respondents gave it a high value, and the difference was not significant between them (Figure 1). Another option is that, when respondents scored the slaughtering method, some might have associated it with the meat appearance in the store or in the butcher’s shop.

The lowest value was given for breed (Figure 1). The differences between hybrids and meat type breeds in terms of meat quality are weak [3]. Thus, the low value is professionally justified.

The role of origin, production system and slaughtering method highly depended on the nationality of the respondents, their country of residence. 

According to our knowledge the impact of gender’s preference on rabbit meat has been hardly investigated. It seems females were more sensitive, so they got higher scores by 0.13–0.45 (*p* < 0.05) for all factors examined than males. The differences were the highest in the case of slaughtering (0.45) and housing methods (0.34), the differences were the smallest regarding origin and breed. In general, females were more sensitive to housing conditions and welfare [42], 55% of females and 27.4% of males considered these very important [43].

The age of respondents affected significantly the scores for origin, feeding method and breed (Table 2), and no significant effects were found in housing condition, slaughtering and level of processing. All factors were considered to be more important by the age group of 30–49 years. The feeding method was also important for ages of 50–59 and the breed for ages 60+. Generally, the youngest generation gave the lowest values. So, it seems that middle-aged people were interested the most where the rabbit meat originates from and under what conditions it was produced, in contrast with the young generation. This is a general attitude of young people regarding meat consumption, not just rabbit meat [44]. Despite of the clear trends, the difference was only significant between the youngest and the other age groups in origin, between the groups of people aged 18–29 and of people 50–59 in breed, with a higher value for the younger group, and between the group of people aged 40–49 years and the youngest or the oldest group in feeding. Szendrő [26] also found that the housing system of rabbits was important for respondents at ages 30–39, and that of the feeding method for people aged 40–49 years. 

There are two options for examining the production method and purchase form depending on the countries. One when comparing the countries in each factor, and the other when comparing the factors in each country. First we evaluated the effect of country on the different factors.

The significantly highest values for origin were given in France, Italy and Hungary (4.45–4.65) followed by Brazil and Poland (also above 4), while the lowest value (2.67) was obtained from the Chinese respondents (Table 3). Feeding was the most important in Poland (4.47), followed by Italy, Hungary and Mexico (3.96–4.27), while the lowest value was given also in China. Housing has been given an important role in Poland (4.36), and also in Italy, Hungary, Mexico and Brazil (4.00–4.27), while it was the least important in France (3.15). The significantly highest scores for the level of processing were given in Brazil and Poland (around 4.40), but the scores were also near 4.0 in China, Mexico and Hungary (3.87–3.94). Significantly lower values were given in Italy and Spain. It was the least important to French respondents (3.30). For the slaughtering method, the highest score was given by the Brazilian respondents (4.40) followed by Mexicans, then by the Italians and the Polish, and the significantly lowest point was given by the French people (3.05). The breed was a less important factor in all countries. The highest value (3.40) was given by Polish respondents, but it was also high in Hungary. In Spain, France and Brazil low scores (2.13–2.33) were given.

Examining the role of factors within countries, the following could be concluded. In Spain, no factor received a higher score than 4, however, the feeding and breed were the most and the least important factors, respectively (Table 3). Spanish researchers evaluated the role of origin and brand in detail [15]. The most valuable origin was the Catalonian (Catalonian identity), followed by Spanish, while the imported rabbit meat was the least preferred.

In Italy the origin, housing condition and feeding were important; the highest value was given for origin, while the breed received a very low value (Table 3).

In France, the origin was considered to be a very important factor (Table 3). Feeding, the level of processing that got much lower scores and the breed was not essential. According to another study consumers paid more attention to the French origin [23], while the information on the welfare of the rabbit breeding was not interesting for respondents. These results are completely consistent with the present investigation.

In Poland, the feeding, level of processing, housing condition and origin received high scores, and among them feeding got the highest ones (Table 3). Respondents’ perception of the production method and purchase form of rabbit meat may partly differ from those of other European countries, because most rabbits originated from backyard farms [13], and perhaps rabbit meat is bought in local markets. So, the differences between Polish and other European respondents could be rooted in the different production systems and markets.

In Hungary, the order of the factors above 4 scores was the origin, feeding and housing. The level of processing and slaughtering method were less important to the respondents, and breed got the lowest score (Table 3). In a former Hungarian study, the housing and feeding also received higher and origin and genotype lower scores [26].

The order of importance of each factor was different in China compared to European countries. All factors received a low score, which could be connected with the high ratio of pork consumption (Chinese are mainly pork-eaters) Although China is the leader country in rabbit production, which has been increasing rapidly [44], rabbit meat has not been a traditional meat, that is, it may be related to a lower appreciation of rabbit meat. The highest score was given for the level of processing and the lowest for origin (Table 3).

In Brazil, the highest scores were given for origin, the level of processing and the slaughtering method. Feeding and housing got a little lower score than 4. In Mexico, the highest scores were given to the slaughtering method, feeding and housing and a little lower to level of processing and origin (Table 3).

The opinion about animal welfare, where the most important factor is housing, was examined in detail in different European countries by a Eurobarometer [45]. According to the respondents, the importance of welfare on a 1–10 scale was the highest in Italy and France (7.8, which is similar to the average in the EU), and the lowest in Hungary (7.3) and Spain (6.8). The knowledge about the housing conditions under which animals are farmed was higher in Poland (77%) and France (75%), and low in Hungary (61%) and Spain (48%). Customers’ willingness for shopping patterns increased due to welfare considerations [46]. It is the highest in Italy and the lowest in Hungary [46]. Higher values were received for female than for male respondents [47].

### 3.2. Purchasing Form

Formerly little emphasis was put on the development of preserved rabbit meat products such as salted or smoked meat, sausages, hamburger, etc. [41]. Currently, the processing industry is moving more and more towards the introduction of modern products (i.e., ready meals, ready-to-cook products, etc.), mainly for the young who want to spend a shorter time in the kitchen preparing food [40]. The same trend can be observed in rabbit processing, which was previously seen in poultry meat products. The proportion of whole carcasses has been decreasing and that of the cut-up and prepared products is increasing [41]. Today, there are several different kinds of rabbit meat products in the supermarkets.

We have not found any literature on the effect of the level of processing on the buying decision of rabbit meat products. Hence, this is a new evaluation.

**Figure 2:**
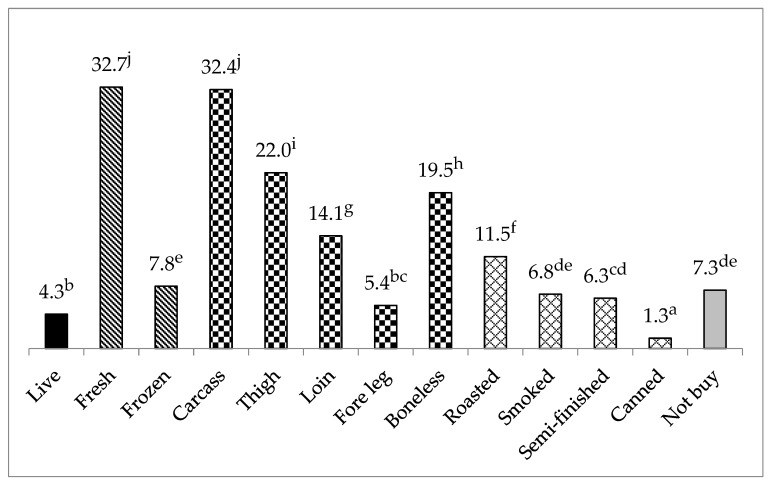
Effect of rabbit meat forms on consumers’ buying decision (%; multiple response option). The columns of live, fresh and frozen, the whole carcass and its parts, the prepared meat products and who do not buy rabbit meat are marked with different patterns. ^a–j^ Means with different superscripts differ at *p* < 0.05.

In general, we found very large and mostly highly significant differences among preferences for different products (Figure 2). It was a multiple choice question; respondents could mark more products. Results show that very few people wanted to buy live rabbits. Presumably they do not want to deal with slaughtering and processing. Thus, it is established that four times more respondents chose fresh meat than frozen. One-third of the respondents preferred the whole carcass, and among the cut-up products the highest percentage was given for thighs, followed by the loin, while the fore leg was not a preferred form of purchase. It should be noted, however, that the relatively favored boneless meat is primarily loin (fillet) since thighs are rarely boned. Roasted meat was the most favored among the listed processed products, and only a few respondents chose the canned product. The percentage of respondents who “would not buy in any case/form” was 7.3%.

#### 3.2.1. Effect of Gender

In the present study a higher rate of male respondents chose different purchasing forms. The differences were significant in the case of fresh meat (male: 36.6% and female: 29.5%), whole carcass (male: 40.1% and female: 26.2%), thigh (male: 25.5% and female: 19.3%), loin (male: 15.8% and female: 12.7%) and roasted meat (male: 14.2% and female: 9.4%). It can be established that meat was perceived more negatively by females than by males, which is based on emotional and moral reasons [17]. To date, the effect of gender on rabbit meat consumption or its preference was evaluated generally, without information about the purchasing form. The meat products and sausage consumption of males exceeded that of females [17,32,48].

#### 3.2.2. Effect of Age

The effect of age on rabbit meat consumption was significant in the case of fresh meat, carcass, thigh, loin and boneless meat (Table 4). The proportion of rabbit meat and rabbit meat product consumption was significantly the smallest among young respondents. This is in connection with the general attitude of the young generation [44]. In addition, the fact that the preparation of rabbit meals takes more time in the kitchen also contributes to lower consumption. Respondents aged 50–59 chose the highest proportion of fresh meat and whole carcass, and there was no significant decrease in the 60+ age group. This may be attributed to the fact that the elderly are traditional rabbit meat consumers. However, by contrast, in a Spanish study rabbit meat consumption frequency was similar between people aged 25 and 54, while it decreased in groups 55–64 and 65–75 years of age [19].

A possible explanation for older people’s consumption of less meat and other foods could be the decline of gustatory and in olfactory functions of the elderly, which may lead to a decrease of the pleasantness of food, which eventually results in a decrease of appetite and food intake [48]. However, meat-eating is very important for pregnant women, babies for developing the brain functions and for the old generation for slowing down the process of mental degradation [49]. However, more and more people, mainly the young, are stressing the harmful or perceived harmful environmental effects of eating meat, its contribution to global warming, welfare issues, the risk of various diseases, moral issues, etc. [44,50,51,52].

#### 3.2.3. Effect of Education Level

The respondents’ attitude depended also on the education level. The higher educated respondents appreciated a semi-finished product 3.1 times, the loin 2.8 times and the boneless meat 2 times more than secondary school graduates (Table 5). There was a slight difference in the whole carcass, while the live rabbits were more preferred by secondary school graduate respondents. It seems that higher educated persons like rabbit meat more and they chose products that are easier to cook. In a Spanish study also the higher educated persons consumed rabbit meat more frequently than those with lower educational qualifications [19]. It is clear that higher educated persons are more demanding of nutritional value and prefer consuming healthy rabbit meat as well.

The connection between income and meat consumption is well documented. There is a very close connection between per-capita GDP and meat consumption [53]. In China a rapid and sustained economic growth has been observed [54], and also the living standards have increased. One result of this is that the Chinese per capita meat consumption increased from 8.3 kg in 1997–1999 to 55.1 kg in 2011–2013 [55]. Of course, rabbit meat production and consumption have increased, but they are still low [56].

#### 3.2.4. Effect of Income Level

The income level of respondents also affected the preference (Table 6). More of those respondents who had the lowest income per household chose rabbit meat less frequently (*p* < 0.05) compared to higher income groups. The only exception was whole carcass, which was the least favored by the wealthiest respondents (*p* < 0.05). The lowest-income group chose mainly whole carcass, while, compared to other groups, the fresh, pieced and boneless meats were the most preferred by people who “live well but can only set little money aside” and in the case of boneless by the wealthiest/the most affluent group as well. There was no significant difference between the other income groups in any of the forms. However, no differences were found between those who would not buy rabbit meat in any case. Escriba-Perez et al. [19] examined the rabbit meat consumption habit of different social groups. A linear increasing tendency was observed in consumption frequency between the low and upper class. A similar observation was made by Szendrő [26] in a former study.

The connection between income and meat consumption is well documented. There is a very close connection between per-capita GDP and meat consumption [53]. In China a rapid and sustained economic growth has been observed [54], and also the living standards have increased. One result of this is that the Chinese per capita meat consumption increased from 8.3 kg in 1997–1999 to 55.1 kg in 2011–2013 [55]. However, rabbit meat production and consumption have increased there, but they are still low [56].

#### 3.2.5. Effect of Nationality

The results in Table 7 are based on multiple response options; therefore, the values show the preference order and are not relative to one another. Compared to the average values presented in Figure 2, a wide variability among countries can be found. According to the respondents, very few people wanted to buy live rabbits. The significantly highest value was in the case of China (8.5%), Poland followed by France, and the lowest in Brazil (1.1%) and Spain (0.4%). Only Mexicans and Brazilians were interested in frozen rabbit meat. In contrast to frozen meat, all countries, except Brazil, preferred fresh meat. The highest ratio was in France (64.2%), but it was above 50% in Mexico and Spain. It is interesting that, compared to the other two Mediterranean countries, this figure was below 40% in Italy. However, in Brazil and China, the preference for fresh rabbit meat was only around 10%. The highest percentage of preference for whole carcass was found in France (62.7%) and Mexico. It seems that traditional cooking is even more important in these countries, but the cheaper carcass prices may also play a role. There was a highly significant difference in the preference for a whole carcass between the two Latin American countries (Mexico: 55.0% and Brazil: 13.9%) and also among the Mediterranean countries (France: 62.7%, Spain: 38.8% and France 29.8%). The least interest was observed among Brazilians.

Among the cut-up products the preference for the thigh, loin and fore leg were evaluated (Table 7). An extremely high preference for the thigh was observed in France (61.2%), and the lowest values were found in Mexico, Brazil and China, however, the thighs were preferred in all European countries. The average preference for loin was lower than that of thigh; however, a preference of 35% was observed in Mexico (*p* < 0.05). Values were above 20% in Spain and Poland while it was only 1% in China. The preference for the fore leg was the highest in France and Spain (around 20%), probably used for some specialty, while in other countries it was between 1.1% and 6.6%. Significantly the highest proportions of Mexican and Italian respondents preferred boneless meat (approximately 40%), while in China this value was only 2.5%

Depending on the local eating habits more and more prepared foods have been produced and purchased in different countries. The preference for roasted, smoked, canned meat and semi-finished products were evaluated (Table 7). A very high preference for roasted meat was found in Mexico (46.7%), while it was only between 5.6% and 7.9% in Italy, Poland and Hungary. Again, there was a difference among the Mediterranean countries. Smoked rabbit meat was also popular in Mexico (33.3%), but hardly known in Italy and Poland. None of the countries were characterized by canned rabbit meat consumption. The highest value of semi-finished products was recorded in Mexico (23.3%), while it was between 7.5% and 13.4% in the Mediterranean countries, and very low values were observed in Poland, Hungary and Brazil. It can be stated that prepared meals were the most popular in Mexico.

Although China is the world’s largest producer of rabbit meat, it still had the highest proportion of non-rabbit consumers, followed by Hungary (Table 7). On the other hand, almost everybody consumed rabbit meat in Italy, France, Brazil and Mexico.

For the Spanish respondents the purchasing form was the most important [15]. The highest weight was assigned to the entire level, followed by pieced and boneless rabbit meat [15]. Results of another questionnaire showed that sliced rabbit was the respondents’ first choice, followed by ready packaged meat and finally by whole carcass. Those who like cooking chose the sliced rabbit more often and the ready packed less often [22]. This order is partially consistent with the results in Table 7. In a former Hungarian questionnaire, the preference order was very similar to the present study except for the loin [24]. In a Chinese study [10] respondents mainly bought fresh meat. Most of the rabbit meat is sold as whole carcass or cut-up parts and it is cooked at home or in restaurants. Lately, several new products have been introduced, like frozen, smoked, roasted, canned, cured, dried, sauce-picked products and even rabbit meat sausage [56]. Respondents rarely bought packed meat and processed products [5].

## 4. Conclusions

As the majority of the questionnaire was spread from universities and research institutes, accordingly, the majority of the respondents had a higher level of education. Hence, both the results and the conclusions drawn were primarily valid for the higher-educated and better-paid part of the society. However, a measurable number of responses were received from each income category.

Although there were many similarities among the three Mediterranean and the two Latin American countries, respondents sometimes had very different views on the consumption of rabbit meat. At the same time, it is not surprising that the opinion of the Chinese differed from other countries. While rabbit origin was the most important factor in Italy and France, in Spain it was rated as moderately important. While Brazilian respondents ranked the origin, slaughtering method and level of processing as the most important factors, Mexicans gave high scores to feeding in addition to slaughter. The level of processing was the only area where there was a significant difference between Polish and Hungarian respondents’ opinion. In China, respondents generally gave low scores, and within them, the role of origin was rated very low, while the level of processing was considered the most important.

Respondents would not like to buy live rabbits in any country. The freshness of the meat was the most important in Spain and France, but not in Italy; however, the frozen meat was not preferred in any Mediterranean countries. The preference of a whole carcass and thigh was much higher in France than in Spain or Italy. The roasted meat was popular in Spain and France, while that of a semi-finished product in Italy and France. Between the two former socialist countries a significant difference was observed only in terms of a whole carcass with higher popularity in Poland. Comparing the Brazilian and the Mexican respondents’ opinion, almost all meat products were more preferred in Mexico. Most of the Chinese respondents gave low values for several products compared to the other countries, except for the smoked products, which were favored in China.

It can be concluded that no general trend for all countries could be detected. Stakeholders willing to export their products should become aware of the expectations of the customers in each country.

## Figures and Tables

**Table 1:** The distribution of the sample.

Description	*n*	%
Total respondents	2205	100
**Gender**		
Female	1233	55.9
Male	972	44.1
**Age, year**		
>18	16	0.7
18–29	743	33.7
30–39	466	21.1
40–49	437	19.8
50–59	364	16.5
60+	179	8.1
**Education background**		
College, university	2005	90,8
Secondary school	187	8.5
Elementary school	13	0.6
**Household income**		
Live very well and with a high enough income to set money aside	382	17.3
Live well but can only set little money aside	1082	49.1
Just enough, but cannot set any money aside	508	23.0
Not enough for a proper living	91	4.1
Have difficulty covering daily expenses	22	1.0
No answer/ Don’t know	120	5.4

**Table 2:** Effect of production condition and purchase form on buying decision depending on the age (on 1–5 scale).

Age, Years	Origin		Breed		Housing		Feeding		Slaughtering		Level of Processing	
	Mean	SD	Mean	SD	Mean	SD	Mean	SD	Mean	SD	Mean	SD
18–29	3.80 ^a^	1.33	2.89 ^b^	1.14	3.96	1.15	3.99 ^a^	1.14	3.72	1.26	4.01	1.13
30–39	4.22 ^b^	1.11	2.73 ^a,b^	1.09	3.86	1.20	4.04 ^a,b^	1.09	3.70	1.33	3.91	1.17
40–49	4.29 ^b^	1.05	2.72 ^a,b^	1.06	4.01	1.19	4.24 ^b^	1.06	3.93	1.16	3.93	1.16
50–59	4.13 ^b^	1.21	2.57 ^a^	1.08	3.89	1.24	4.11 ^a,b^	1.08	3.75	1.28	3.75	1.28
60+	4.13 ^b^	1.27	2.78 ^a,b^	1.22	3.73	1.24	3.88 ^a^	1.22	3.81	1.15	3.81	1.15
Prob.	<0.001		0.048		0.097		0.008		0.281		0.074	

^a,b^ Means with different superscripts in a column differ (*p* < 0.05).

**Table 3:** Effect of the production method and slaughtering method and processing on purchase decision depending on the countries (on 1–5 scale).

	Origin	Breed	Housing	Feeding	Slaughtering	Level of Processing
Spain						
*n*	199	181	196	202	195	197
Mean	3.45 ^b^	2.13 ^a^	3.42 ^a^	3.8 ^a,b^	3.50 ^a,b,c^	3.50 ^a^
SD	1.31	1.10	1.27	1.19	1.30	1.19
Italy						
*n*	206	185	205	204	198	193
Mean	4.50 ^d^	2.50 ^bc^	4.27 ^c,d^	4.22 ^c,d^	3.68 ^b,c,d^	3.76 ^a,b^
SD	0.94	1.17	1.04	1.02	1.25	1.23
France						
*n*	65	61	65	65	65	63
Mean	4.65 ^d^	2.23 ^ab^	3.15 ^a^	3.66 ^a,b^	3.05 ^a^	3.30 ^a^
SD	0.74	1.15	1.41	1.25	1.36	1.45
Poland						
*n*	146	136	148	152	144	143
Mean	4.29 ^c,d^	3.40 ^e^	4.36 ^d^	4.47 ^d^	3.79 ^c,d^	4.38 ^d^
SD	0.92	1.19	0.84	0.76	1.27	0.82
Hungary						
*n*	313	296	309	312	304	309
Mean	4.45 ^d^	3.15 ^d,e^	4.01 ^c,d^	4.29 ^c,d^	3.64 ^b,c^	3.87 ^b^
SD	0.97	1.26	1.10	0.96	1.28	1.15
China						
*n*	113	113	120	119	117	123
Mean	2.67 ^a^	2.89 ^c,d^	3.56 ^a,b^	3.54 ^a^	3.29 ^a,b^	3.94 ^b,c^
SD	1.21	1.13	1.13	1.15	1.13	1.08
Brazil						
*n*	106	98	108	105	106	105
Mean	4.39 ^c,d^	2.33 ^a,b^	3.96 ^b,c,d^	3.91 ^a,c^	4.40 ^e^	4.40 ^c,d^
SD	1.06	1.24	1.23	1.24	1.09	1.02
Mexico						
*n*	54	56	55	55	55	54
Mean	3.85 ^b,c^	2.77 ^b,c,d^	4.00 ^b,c,d^	4.16 ^b,c,d^	4.22 ^d,e^	3.89 ^b,c^
SD	1.34	1.28	1.11	1.07	1.15	1.24
Other countries						
*n*	312	297	315	317	309	310
Mean	4.13 ^c^	2.78 ^c^	3.97 ^c^	4.05 ^b,c^	3.80 ^c,d^	4.00 ^c^
SD	1.20	1.37	1.25	1.17	1.32	1.15
Prob.	<0.001	<0.001	<0.001	<0.001	<0.001	<0.001

^a–e^ Means with different superscripts in a column differ (*p* < 0.05).

**Table 4:** Effect of purchase forms on the respondents’ attitude (%) depending on the age with multiple response options.

Purchasing Form	Level of Income				Prob.
	1*	2*	3*	4*	
**Live**	7.7	5.7	3.9	2.4	0.059
Fresh	18.7 ^a^	29.5 ^b^	36.4 ^c^	32.5 ^b,c^	0.001
Frozen	6.6	6.5	8.9	8.6	0.549
Whole carcass	30.8 ^a,b^	31.5 ^b^	36.3 ^b^	24.9 ^a^	0.001
***Cut-up products***					
Pieced	11.0 ^a^	20.1 ^b^	27.0 ^c^	17.5 ^a,b^	<0.001
Thigh	17.6	19.9	24.7	22.8	0.131
Loin	3.3 ^a^	14.0 ^b^	16.1 ^b^	13.6 ^b^	0.009
Fore leg	0.0	5.1	6.5	5.0	0.110
Boneless	6.6 ^a^	16.1 ^b^	22.0 ^c^	22.8 ^c^	<0.001
***Prepared food***					
Roasted	15.4	12.2	11.7	10.5	0.308
Prepared food	6.6	10.0	12.5	10.5	0.320
Smoked	7.7	8.5	6.7	6.0	0.397
Canned	1.1	0.8	1.7	1.3	0.669
Semi-finished product	5.5	7.3	6.3	6.3	0.679
*I would not buy in any case*	7.7	6.9	6.6	7.6	0.944

1*—Not enough for a proper living; 2*—Just enough, but cannot set any money aside; 3*—Live well but can only set little money aside; 4*—Live very well and with a high enough income to set money aside. ^a–c^ Means with different superscripts in a row differ (*p* < 0.05).

**Table 5:** Effect of purchase forms on the respondents’ attitude (%) depending on the education level with multiple response options.

Purchasing Form	Education Level		Prob.
	Secondary School	Higher Education	
**Live**	4.8	4.0	<0.001
Fresh	25.7	33.2	0.070
Frozen	4.3	8.1	0.191
Whole carcass	28.9	32.4	0.005
***Cut-up products***			
Pieced	15.0	23.4	0.058
Thigh	15.0	22.7	0.105
Loin	5.3	14.9	0.001
Fore leg	4.3	5.5	0.160
Boneless	10.2	20.4	0.007
***Prepared food***			
Roasted	8.0	11.8	0.264
Prepared food	3.7	11.7	0.004
Smoked	4.8	7.0	0.667
Canned	0.0	1.4	0.411
Semi-finished product	2.1	6.6	0.048
*I would not buy in any case*	10.7	7.0	0.061

**Table 6:** Effect of purchase forms on the respondents’ attitude depending on the income with multiple response options.

Purchasing Form	Level of Income				Prob.
	1*	2*	3*	4*	
**Live**	7.7	5.7	3.9	2.4	0.059
Fresh	18.7 ^a^	29.5 ^b^	36.4 ^c^	32.5 ^b,c^	0.001
Frozen	6.6	6.5	8.9	8.6	0.549
Whole carcass	30.8 ^a,b^	31.5 ^b^	36.3 ^b^	24.9 ^a^	0.001
***Cut-up products***					
Pieced	11.0 ^a^	20.1 ^b^	27.0 ^c^	17.5 ^a^^,^^b^	<0.001
Thigh	17.6	19.9	24.7	22.8	0.131
Loin	3.3 ^a^	14.0 ^b^	16.1 ^b^	13.6 ^b^	0.009
Fore leg	0.0	5.1	6.5	5.0	0.110
Boneless	6.6 ^a^	16.1 ^b^	22.0 ^c^	22.8 ^c^	<0.001
***Prepared food***					
Roasted	15.4	12.2	11.7	10.5	0.308
Prepared food	6.6	10.0	12.5	10.5	0.320
Smoked	7.7	8.5	6.7	6.0	0.397
Canned	1.1	0.8	1.7	1.3	0.669
Semi-finished product	5.5	7.3	6.3	6.3	0.679
*I would not buy in any case*	7.7	6.9	6.6	7.6	0.944

1*—Not enough for a proper living; 2*—Just enough, but cannot set any money aside; 3*—Live well but can only set little money aside; 4*—Live very well and with a high enough income to set money aside. ^a–c^ Means with different superscripts in a row differ at *p* < 0.05 level.

**Table 7:** Effect of purchase forms on the respondents’ attitude depending on the countries with multiple response options.

Purchasing Form	Country									Prob.
	Sp	It	Fr	Pl	Hu	Ch	Br	Mx	Others	
**Live**	0.4^a^	3.3 ^b,c^	4.5 ^c,d^	6.1^c,d^	2.9 ^b,c^	8.5^d^	1.1^a,b^	3.3 ^b,c^	8.1 ^d^	<0.001
Fresh	50.2 ^d^	38.0 ^c^	64.2 ^e^	36.4 ^b,c^	41.7 ^c^	8.0 ^a^	12.5^a^	55.0^d,e^	30.2 ^b^	<0.001
Frozen	4.4 ^a,b^	3.3 ^a,b^	3.0 ^a,b^	6.6 ^b^	5.7 ^b^	2.0 ^a^	18.1^c^	21.7 ^c^	7.7 ^b^	<0.001
Whole carcass	38.8 ^c^	29.8 ^b^	62.7 ^d^	49.0 ^d^	28.8 ^b^	22.9 ^b^	13.9^a^	55.0 ^d^	38.4 ^c^	<0.001
***Cut-up products***										
Pieced	59.0 ^d^	42.6 ^c^	11.9 ^b^	14.1 ^b^	21.9 ^b^	3.0 ^a^	12.8^b^	41.7 ^c^	13.3 ^b^	<0.001
Thigh	30.0 ^c^	21.5 ^b^	61.2 ^d^	20.2 ^a,b^	33.3 ^c^	12.9 ^a^	13.6^a^	10.0 ^a^	14.9 ^a^	<0.001
Loin	21.6 ^d^	13.2 ^c^	17.9^cd^	22.7 ^d,e^	17.9^c,d^	1.0 ^a^	11.4^b,c^	35.0 ^e^	7.7 ^b^	<0.001
Fore leg	18.1 ^d^	6.6 ^c^	20.9 ^d^	2.0 ^a,b^	5.0 ^b,c^	3.5 ^a,b,c^	1.1 ^a^	3.3 ^a,b,c^	2.6 ^a,b^	<0.001
Boneless	28.2 ^c^	38.8 ^d^	10.4 ^b^	12.6 ^b^	23.6 ^c^	2.5 ^a^	15.6 ^b^	41.7 ^d^	13.0 ^b^	<0.001
***Prepared food***										
Roasted	15.9 ^c,d^	6.2 ^a^	16.4 ^c,d^	5.6 ^a^	7.9 ^a,b^	17.4 ^d^	11.4 ^b,c^	46.7 ^e^	10.2 ^a,b^	<0.001
Prepared food	11.9 ^a,b^	10.7^a,b^	19.4 ^b^	10.1 ^a^	8.3 ^a^	9.0 ^a^	9.4 ^a^	45.0 ^c^	10.2 ^a^	<0.001
Smoked	4.4 ^a,b,c^	2.1 ^a^	6.0 ^a,b,c,d^	3.5 ^a,b^	7.4 ^b,c,d^	10.0 ^d^	8.6 ^c,d^	33.3 ^e^	5.3 ^b,c^	<0.001
Canned	2.2 ^c,d^	0.8 ^a,b,c^	6.0 ^d^	2.0 ^b,c,d^	0.5 ^a,b^	1.0 ^a,b,c^	0.3 ^a^	3.3 ^c,d^	1.4 ^a,b,c^	0.005
Semi-finished product	7.5 ^b,c^	13.2^d,e^	13.4^c,d,e^	3.5 ^a,b^	3.1 ^a^	8.5 ^c,d^	4.2 ^a,b^	23.3 ^e^	3.5 ^a^	<0.001
*I would not buy in any case*	4.0 ^b^	2.9 ^b^	1.5 ^b^	6.6 ^b,c^	9.5 ^c,e^	13.4 ^e^	3.3 ^b^	0.0 ^a^	12.3 ^e^	<0.001

Note: Sp = Spain, It = Italy, Fr = France, Pl = Poland, Hu = Hungary, Ch = China, Br = Brazil, Mx = Mexico. ^a–e^ Means with different superscripts in a row differ at *p* < 0.05 level.
